# Longitudinal Household Trends in Access to Improved Water Sources and Sanitation in Chi Linh Town, Hai Duong Province, Viet Nam and Associated Factors

**DOI:** 10.3934/publichealth.2016.4.880

**Published:** 2016-10-24

**Authors:** Tran Thi Tuyet-Hanh, Tran Khanh Long, Hoang Van Minh, Le Thi Thanh Huong

**Affiliations:** Hanoi University of Public Health, 1A Duc Thang Road, Duc Thang Ward, North Tu Liem District, Hanoi, Viet Nam

**Keywords:** improved water sources, improved sanitation, trend, CHILILAB, Vietnam

## Abstract

**Objective:**

This study aims to characterize household trends in access to improved water sources and sanitaton in Chi Linh Town, Hai Duong Province, Vietnam, and to identify factors affecting those trends.

**Method:**

Data were extracted from the Chi Linh Health and Demographic Surveillance System (CHILILAB HDSS) database from 2004–2014, which included household access to improved water sources, household access to improved sanitation, and household demographic data. Descriptive statistical analysis and multinominal logistic regression were used. The results showed that over a 10-year period (2004–2014), the proportion of households with access to improved water and improved sanitation increased by 3.7% and 28.3%, respectively. As such, the 2015 Millennium Development Goal targets for safe drinking water and basic sanitation were met. However, 13.5% of households still had unimproved water and sanitation. People who are retired, work in trade or services, or other occupations were 1.49, 1.97, and 1.34 times more likely to have access to improved water and sanitation facilities than farming households, respectively (*p* < 0.001). Households living in urban areas were 1.84 times more likely than those living in rural areas to have access to improved water sources and improved sanitation facilities (OR =1.84; 95% CI = 1.73–1.96). Non-poor households were 2.12 times more likely to have access to improved water sources and improved sanitation facilities compared to the poor group (OR = 2.12; 95% CI = 2.00–2.25). More efforts are required to increase household access to both improved water and sanitation in Chi Linh Town, focusing on the 13.5% of households currently without access. Similar to situations observed elsewhere in Vietnam and other low- and middle- income countries, there is a need to address socio-economic factors that are associated with inadequate access to improved water sources and sanitation facilities.

## 1. Introduction

Access to safe water and basic sanitation is needed to maintain and improve health [1]. Polluted water and lack of sanitation increase the risk for various diseases, including cholera, typhoid, schistosomiasis, respiratory infections, skin infections, eye infections, and even some cancers through exposure to carcinogens [2]–[4]. The target of the Millennium Development Goal (MDG) 7.C aimed to reduce the proportion of people without sustainable access to safe drinking water and basic sanitation by half between 1990 and 2015. However, there are still currently more than 700 million people, mostly the poor and marginalized people in low- and middle- income countries, that lack access to improved sources of drinking water [5]. Access to improved sanitation is also a substantial problem for low- and middle- income countries where approximately 2.5 billion people do not use any improved toilets, with one billion people still practicing open defecation [6].

A study by Tuyet-Hanh et al. (2016) found that access to both improved water and sanitation in Vietnam at the national level increased significantly from 2000 to 2011, and met the MDG's target 7.C for water and sanitation. Location and socio-economic characteristics including region, living area, wealth index, and the household head's characteristics (ethnicity, gender, and educational levels) had a strong association with access to improved water and sanitation facilities [7]. For example, report by the General Statistics Office of Vietnam and UNICEF showed that by 2014, 92% and 79% of the population in Vietnam had access to improved sources of drinking water and sanitation, respectively [8]. Yet, disparities exist between ethnic minority and Kinh households, with only 75% of ethnic minority households having access to improved drinking water source compared to 95% of Kinh households. Furthermore, 47% of ethnic minority population did not have access to sanitation facilities. When comparing urban-rural locations, more than 90% of urban populations had access to sanitation facilities compared to 73% in rural areas.

Chi Linh was a rural district in Hai Duong Province in the Red River Delta region of Vietnam, which is approximately 80 km from Hanoi Capital City. Both Chi Linh District and Hai Duong Province developed and urbanized very rapidly to become Chi Linh Town, and Hai Duong City, respectively. The Chi Linh Health and Demographic Surveillance System (CHILILAB HDSS) was established in 2003 by the Hanoi School of Public Health. This surveillance system was designed as a site for field training, operational research, and developing and implementing health interventions. CHILILAB HDSS collected demographic information at both the household and individual level, and information on water and sanitation status at the household level. This article analyzes the survey data from 2004–2014 to describe household trends in access to improved water and sanitation separately, and to identify factors associated with access to improved water and sanitation facilities in combination, in Chi Linh Town. The results will help to draw a comprehensive picture of the water and sanitation situation at local level, and inform policymakers about the direction that adequate access to improved water sources and sanitation is heading.

## 2. Materials and Methods

### 2.1. Data Source

**Figure 1:**
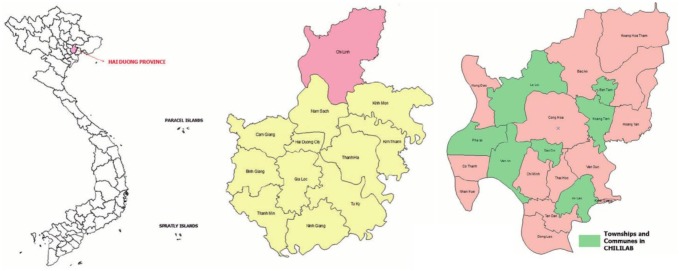
Maps of Vietnam, Hai Duong Province and Chi Linh Town [9].

The study used secondary data from CHILILAB HDSS database. This epidemiological and population surveillance system was established by Hanoi School of Public Health in 2003 in seven communes/towns of Chi Linh Town, Hai Duong Province. Data collected through this system consisted of basic demographic information at both household and individual levels, and a list of the households in the district was made at the time of the original baseline survey. The baseline survey was repeated biennially. General household information, morbidity data, general household socio-economic and demographic indicators were collected. Entire populations of three selected townships and four communes within the Chi Linh Town were invited to participate in the study (Figure 1) [9]. From the beginning, CHILILAB HDSS included 58,761 participants from 16,019 households. Up until 2014, this system covered 59,581 participants from 16,971 households. This study used data extracted from 2004 to 2014 from the HDSS, including basic information of household; location (urban or rural); number of persons living in the house; and information related to water and sanitation use. In addition, demographic information was extracted from the head of household including age, occupation, gender, location, economic status (collected through the household poor certificate of the council, except in 2004), marital status, education level, ethnic, and religion.

### 2.2. Variables

The improved water and sanitation sources of household was defined according to WHO/UNICEF Joint Monitoring Programme [10]. The dependent variable of this article was the combined status of improved water and improved sanitation used in the household. An improved water and sanitation status meant that a household used both improved water source and improved sanitation condition. All other combinations of water and sanitation condition including improved water source and unimproved toilet; unimproved water source and improved toilet; unimproved water and unimproved toilet were coded as unimproved water and sanitation.

Independent variables in this article were: (1) demographic information of the household including number of family members in household, socio-economic status and location; (2) demographic information of head of household including age, gender, marriage status, occupation, education level, ethic, religion; and (3) time, indicating the period of the baseline survey.

### 2.3. Statistical Analysis

Descriptive statistical analysis were performed using Stata 13. Chi-square test was used to compare between groups. Binary logistic regression modeling using generalized estimating equations (GEE) method was used to identify the association between factors and the status of water and sanitation used in household between 2006 and 2014 (2004 was not included in the analysis because of the lack information relating to the household's economic status in 2004). Significance at *p* < 0.05 was used in all of the computations.

### 2.4. Ethical Approval

The CHILILAB HDSS was approved by the Institutional Review Board at the Hanoi School of Public Health in 2004.

## 3. Results

### 3.1. Household Trends in the Access to Water Sources and Sanitation Facilities in Chi Linh Town, Vietnam, 2004 to 2014

**Figure 2.**
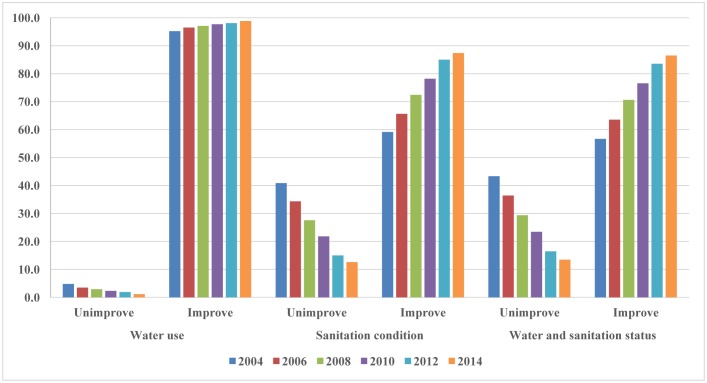
Status of households having access to improved water use and sanitation conditions in Chi Linh Town, 2004–2014.

The results presented in Figure 2 show that during the studied period (2004–2014), the proportions of households having access to improved water sources were high and increased from 95.2% to 98.9%. However, the proportions of households with access to improved sanitation facilities were still lower than those with access to improved water sources, from 59.1% in 2004 to 87.4% in 2014. If considering the situation of households having both improved water and sanitation, the proportions were lower, started from 56.7% in 2004 and steadily increasing to 86.5% in 2014 (*χ*^2^ = 5630.9, *df* = 5, *p* < 0.001).

### 3.2. Access to Improved Water Sources and Improved Sanitation Facilities in Combination in Urban and Rural Areas

As described above, during the period from 2004 to 2014, an increasing trend was observed in households with access to improved water sources and improved sanitation facilities in Chi Linh District. However, as shown in Table 1, the proportions were significantly higher in urban areas compared rural areas (*χ*^2^ = 5630, *df* = 5, *p* < 0.001). For urban areas, the proportions of households with access to unimproved water and unimproved sanitation in the four surveys decreased from 36.6% in 2004 to 6.1% in 2014. For rural areas, the proportions were higher, decreasing from 51.2% to 21.7% over the same time period. Thus, by 2014, the proportion of households having access to both unimproved water and sanitation among rural areas was more than three times higher compared to urban areas (*χ*^2^ = 5630, *df* = 5, *p* < 0.001).

**Table 1.** Access to improved water sources and improved sanitation facilities in combination in urban and rural areas.

		2004	2006	2008	2010	2012	2014
Urban	Unimproved	36.6%	27.3%	19.0%	13.3%	8.0%	6.1%
	Improved	63.4%	72.7%	81.0%	86.7%	92.0%	93.9%
Rural	Unimproved	51.2%	47.0%	41.3%	35.0%	25.8%	21.7%
	Improved	48.8%	53.0%	58.7%	65.0%	74.2%	78.3%
Total	Unimproved	43.3%	36.4%	29.4%	23.5%	16.4%	13.5%
	Improved	56.7%	63.6%	70.6%	76.5%	83.6%	86.5%

The proportion of households in urban areas with access to improved water sources and improved sanitation increased by 30.5% while that in the rural areas increased by 29.5% during the period from 2004 to 2014. By 2014, the proportion in rural areas with access to improved water sources and improved sanitation (78.3%) was still significantly lower than that in urban areas in 2008 and the following years (81% to 93.9) (*p* < 0.001). It, however, should be noted that while rural area access was lower, the percentage changes during this period were very similar between urban areas (30.5% increase) and rural areas (29.5% increase).

### 3.3. Factors Associated with Access to Improved Water Sources and Improved Sanitation

Table 2 presents the results of the multivariate logistic regression, which showed that different factors were strongly associated with the access to improved water sources and sanitation facilities in combination. For example, female head of households were 1.14 times more likely than male head of households to have access to improved water sources and improved sanitation (OR = 1.14; 95% CI = 1.06–1.23). Households living in urban areas were 1.84 times more likely than those living in rural areas to have access to improved water sources and improved sanitation facilities (OR = 1.84; 95% CI = 1.73–1.96). Non-poor households were 2.12 times more likely to have access to improved water sources and improved sanitation facilities compared to the poor groups (OR = 2.12; 95% CI = 2.00–2.25). There were stark differences among education levels of heads of households. Heads of households having secondary, college or upper college educational levels significantly were more likely than those with under secondary grade education to have access to improved water sources and improved sanitation facilities (*p* < 0.001).

**Table 2.** GEE binary logistic regression of factors associated with household access to improved water sources and sanitation facilities in combination ^a^, 2006–2014.

		OR	95% CI
Gender of household's head	Female	1.14	1.06–1.23
	Male	1
Age of household's head		1.02	1.02–1.02
Number of member in household		1.08	1.07–1.1
Location	Urban	1.84	1.73–1.96
	Rural	1
Economic status ^#^	Poor	2.12	2–2.25
	Not poor	1
Marital status of household's head	Living with husband/wife	2.11	1.79–2.49
	Divorce	1.30	1.1–1.54
	Single	1
Occupation of household's head	Retired	1.49	1.36–1.63
	Trade and services	1.97	1.84–2.11
	Others	1.34	1.27–1.41
	Farmer	1
Education of household's head	Secondary	1.69	1.54–1.86
	College	2.50	2.29–2.73
	Upper college	5.31	4.72–5.98
	Under secondary	1
Ethnic	Kinh	1.25	0.83–1.88
	Non-Kinh	1
Religion	Religious	0.71	0.46–1.11
	Non-religious	1

^a^: Note: reference category is unimproved water and sanitation.

^#^: Vietnam has a system of monitoring and certifying the economic status of households. A poor family could be assisted by different government's policies, therefore, a family has the poor certificate could be considered as poor economic status [11].

The occupation of head of households was also associated with the status of access to improved water and sanitation. People who were retired, doing trade and services, or working other occupations were 1.49, 1.97, and 1.34 times more likely to have access to improved water and sanitation facilities than households whose heads being farmers, respectively (*p* < 0.001). Table 2 also shows that Kinh ethnic people were more likely to have access to improved water sources and sanitation facilities than ethnic minority groups, however, this difference was not statistically significant (OR = 1.25; 95% CI = 0.83–1.88). There was no statistically significant difference in access to improved water sources and sanitation facilities between different religious groups.

## 4. Discussion

Based on our findings, great progress was made in providing access to improved water and sanitation facilities in Chi Linh Town from 2004 to 2014. The overall data showed that over a 10-year period (2004–2014), the proportion of households with access to improved water and improved sanitation facilities increased to 98.9% and 87.4%, respectively. Thus, the proportion of households with access to improved sanitation was lower than households with access to improved water. A similar trend was observed at a national level in which the access to improved water and sanitation in 2014 was 92% and 79% [8], respectively, which met the MDG's target for water and sanitation (88% and 75% by 2015) [6]. The overall level of access to improved sanitation facilities in Chi Linh District in 2014 was 87.4%, which was higher than those from Eastern Asia (67%) and the rest of the world (64%) [7],[12]. The proportion of Chi Linh population having both improved water and sanitation in 2014 was 86.5%, which was lower than the population in the Red River Delta in 2011 (94.4%), but higher than the situation in the whole country in 2011 (73.8%) [7]. However, the distribution of households with access to both improved water sources and improved sanitation varied across educational levels, occupation, marital status, year of surveys, living areas, and economical status. A recent study by Tuyet-Hanh et al. (2016) also showed similar results, for example, the distributions of households with access to both improved water and sanitation significantly varied across six regions of Vietnam, urban and rural areas, ethnic groups, five wealth index quintiles, and education levels (*p* < 0.001) [7].

It is important to note that 13.5% of households in the Town in 2014 still had no access to both improved water sources and improved sanitation, despite Hai Duong Province being one of the four provinces to receive the Vietnam Red River Delta Rural Water Supply and Sanitation Project by the World Bank. The project started from 15^th^ September 2005 and closed on 30^th^ June 2013, with the total project cost amounting to US $ 50.12 million for the four provinces [13]. In Hai Duong, a total of 38 communes (included two communes in Chi Linh Town) were included in this project which aimed to provide access to clean water for approximately 200,000 local residents. The impacts of water scarcity and poor access to improved sanitation facilities are well recognized for causing various infectious diseases [14]. Diarrhea, schistosomiasis, trachoma, and intestinal helminthes can be caused by unsafe water, poor sanitation, and the lack of hygiene [15]. Improving access to clean water and sanitation is essential in reducing the incidence of these diseases. For example, Esrey et al. (1991) showed that in settings where the preexisting conditions were poor, a reasonably well-implemented intervention in one or more aspects of hygiene, sanitation, water supply or water quality reduced the prevalence of diarrheal diseases up to one third [16].

Although this study provides a comprehensive analysis on the current situation of the access to improved water sources and sanitation in Chi Linh District, it has a limited amount of data; for example, no data on poor families were available in 2004 for the multivariate logistic regression model. Socio-economic status was also not used since the indicators were not consistent among surveys. Furthermore, this study only analyzed data from Chi Linh District, Hai Duong Province only, which is not representative for the situation in Vietnam. In addition, the safe management of water sources was not included in the surveys. As Bain et al. (2014) reported, improved sources have been shown to be contaminated with feces and 1.8 billion people drink water from contaminated sources [17]. Another study concluded that access to an “improved source” provides a measure of sanitary protection but does not ensure that water is free of fecal contamination nor is it consistent between source types or settings [18]. In Shields et al. (2015), stored water was significantly more likely to be contaminated if it came from a non-piped source [19]. In Vietnam, if considering the quality of the water sources, in 2013 only 38.7% of households had access to water sources that met water quality standard issued by Ministry of Health (QCVN02-2009, MOH) [20]. Thus, when interpreting the results reported in this study as well as results reported in Viet Nam Multiple Indicator Cluster Surveys by General Statistics Office and UNICEF, one should keep in mind that if considering the quality of the water sources according to Ministry of Health's standards, then the percentages of households having access to clean water were much lower than those with access to improved water sources.

## 5. Conclusions and Recommendations

Access to both improved water and sanitation in Chi Linh District of Hai Duong Province in Vietnam increased from 2004 to 2014, and met the MDG's target 7.C for water and sanitation. Living areas, economic status and specific characteristics of the household heads were associated with access to improved water and sanitation facilities. The results described in this article suggest that more efforts should be made to increase the access to improved water sources and sanitation facilities in households of underserved populations, especially those living in the rural communes of Chi Linh District. Furthermore, water and sanitation programs in general should have more focus on increasing access to improved sanitation as the results of this study and other studies showed that the proportions of households having access to improved sanitation were often significantly lower than those with access to improved water. There is also a need to address the socio-economic factors that are associated with inadequate access to improved water and sanitation facilities in Chi Linh District and to increase the proportion of households having access to both improved water and sanitation facilities. This will help to ensure equal access to basic needs, and can play an important role in the prevention and reduction of the burden of waterborne diseases and other diseases with fecal-oral transmission in the studied areas.
